# BPC-157 and Its Novel Hybrid Analogs as Inhibitors of Acetylcholinesterase

**DOI:** 10.3390/ijms27114984

**Published:** 2026-05-30

**Authors:** Juliana Jelińska, Michalina Józwiak, Łukasz Szeleszczuk, Karol Sikora, Wojciech Kamysz, Patrycja Kleczkowska, Marcin Gackowski, Błażej Grodner

**Affiliations:** 1 Department of Biochemistry and Pharmacogenomics, Medical University of Warsaw, 1 Banacha Str., 02-097 Warsaw, Poland; s086037@student.wum.edu.pl; 2 Maria Skłodowska-Curie Medical Academy in Warsaw, al. Solidarności 12, 03-411 Warsaw, Poland; michalina1395@interia.pl; 3 Department of Organic and Physical Chemistry, Medical University of Warsaw, 1 Banacha Str., 02-097 Warsaw, Poland; lukasz.szeleszczuk@wum.edu.pl; 4 Department of Inorganic Chemistry, Faculty of Pharmacy, Medical University of Gdansk, 80-416 Gdansk, Poland; karol.sikora@gumed.edu.pl (K.S.); kamysz@gumed.edu.pl (W.K.); 5 Department of Nursing and Other Health Professions, Center of Postgraduate Medical Education, 01-826 Warsaw, Poland; hazufiel@wp.pl; 6 Department of Toxicology and Bromatology, L. Rydygier Collegium Medicum in Bydgoszcz, Nicolaus Copernicus University in Torun, 2 Jurasza Str., 85-089 Bydgoszcz, Poland; marcin.gackowski@cm.umk.pl

**Keywords:** acetylcholinesterase inhibition, BPC-157, peptide-based inhibitors, hybrid peptides, enzyme kinetics

## Abstract

Acetylcholinesterase (AChE) inhibition remains a key therapeutic strategy in the management of neurodegenerative disorders such as Alzheimer’s disease. In this study, the inhibitory potential of the gastric pentadecapeptide BPC-157 and two newly designed hybrid analogs, CIARA-1 and CIARA-2, was investigated for the first time. The hybrid peptides were rationally designed by combining a BPC-157-derived fragment with an arginine-containing C-terminal sequence to enhance interactions with the enzyme’s active and peripheral binding sites. Enzyme kinetics were evaluated using a modified Ellman assay, and inhibition parameters were determined through Lineweaver–Burk analysis. All tested compounds exhibited a competitive mechanism of inhibition, as evidenced by increased Michaelis–Menten constant (*K_m_*) values with unchanged maximum velocity (*V*_max_), indicating competition with the substrate at the catalytic site of AChE. Among the tested compounds, CIARA-1 demonstrated the highest inhibitory potency, reflected by the lowest inhibition constant (*K_i_* = 0.24 mM) and IC_50_ value (2.52 mM), followed by CIARA-2 (*K_i_* = 0.29 mM; IC_50_ = 2.73 mM) and BPC-157 (*K_i_* = 0.48 mM; IC_50_ = 2.80 mM). These findings were consistent with molecular modeling predictions, supporting stronger binding interactions for CIARA-1. Despite significantly lower potency compared to clinically used AChE inhibitors, the studied peptides represent a promising scaffold for further optimization. Overall, this work demonstrates that BPC-157 and its hybrid analogs act as reversible competitive AChE inhibitors, with enhanced activity observed for structurally modified derivatives. The results highlight the potential of peptide-based hybrid molecules as multifunctional candidates in the development of novel therapeutics targeting cholinergic dysfunction.

## 1. Introduction

Acetylcholinesterase (AChE) is a key enzyme responsible for the hydrolysis of acetylcholine in cholinergic synapses, thereby regulating neurotransmission in both the central and peripheral nervous systems [1,2]. Due to its crucial role in maintaining cholinergic balance, AChE has long been recognized as an important pharmacological target, particularly in the treatment of neurodegenerative disorders such as Alzheimer’s disease, where cholinergic deficits are strongly associated with cognitive decline [3,4].

Inhibition of AChE remains a well-established therapeutic strategy to increase acetylcholine levels in the synaptic cleft and improve cognitive function [5]. Although clinically used inhibitors, such as donepezil and rivastigmine, demonstrate significant efficacy, their therapeutic use is limited by adverse effects and incomplete long-term outcomes [1,6,7,8]. Therefore, the development of novel AChE inhibitors with improved pharmacological properties remains an important objective.

**Figure 1:**
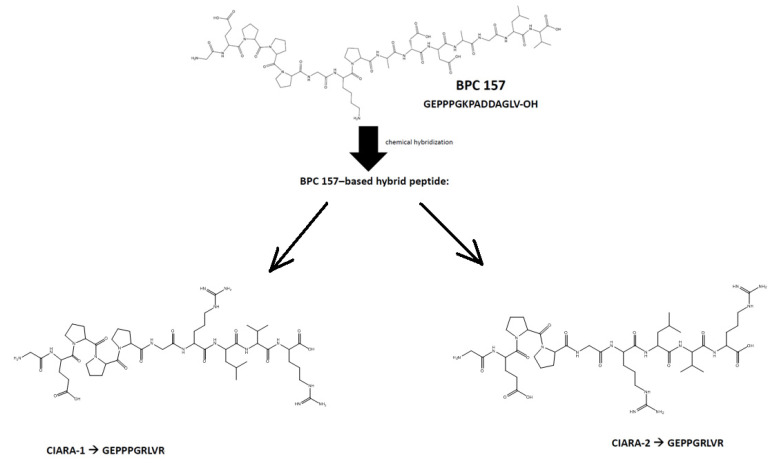
Chemical structures of CIARA-1 (GEPPPGRLVR-OH), CIARA-2 (GEPPGRLVR-OH), and BPC-157 (GEPPPGKPADDAGLV-OH) peptides.

BPC-157, a stable gastric pentadecapeptide (Figure 1), has been extensively studied for its cytoprotective, anti-inflammatory, and regenerative effects [9,10,11]. Importantly, it has also been shown to preserve neuromuscular function by stabilizing acetylcholine receptors and nerve terminals at the neuromuscular junction [12,13], suggesting its role in modulating cholinergic signaling. However, despite these observations, its potential to directly affect acetylcholinesterase activity has not been previously investigated.

To address this gap, BPC-157 was evaluated for its ability to inhibit AChE activity. In addition, novel hybrid peptide analogs, CIARA-1 and CIARA-2, were designed to develop multifunctional molecules (Figure 1). These compounds incorporate a BPC-157-derived fragment at the N-terminus, with variations in proline content that may influence conformational flexibility and binding properties, and a C-terminal arginine-containing sequence (GRLVR-OH). The presence of arginine residues within this fragment may facilitate electrostatic and hydrogen-bonding interactions with the active or peripheral binding sites of AChE and potentially enhance binding affinity compared to the parent peptide. This design strategy reflects an intentional effort to create molecules that not only inhibit AChE but also preserve or enhance the cytoprotective, anti-inflammatory, and regenerative properties of BPC-157.

Recently, increasing attention has been directed toward peptide-based and multifunctional acetylcholinesterase inhibitors as potential therapeutic tools for Alzheimer’s disease [14,15]. In contrast to classical small-molecule inhibitors such as donepezil, rivastigmine, and galantamine [16,17], peptide-derived compounds may offer improved selectivity, reduced off-target toxicity, and the possibility of simultaneously interacting with multiple functional regions of AChE, including both the catalytic active site (CAS) and the peripheral anionic site (PAS) [18]. Such multitarget-directed approaches are considered particularly promising in Alzheimer’s disease due to the multifactorial nature of neurodegeneration [14].

Recent studies have demonstrated that rational modification of peptide sequences and hybrid molecular design can significantly influence AChE binding affinity and biological activity [19]. Peptide-based inhibitors and hybrid ligands that combine cholinesterase inhibition with additional neuroprotective or anti-aggregation properties are increasingly being investigated as multifunctional candidates for Alzheimer’s disease therapy [15]. In particular, structure–activity relationship studies indicate that positively charged residues, conformationally restricted motifs, and hybrid peptide architectures may enhance interactions within the AChE catalytic gorge and peripheral binding regions [18,19].

In this context, the design of CIARA-1 and CIARA-2 was intended not only to explore their inhibitory potential against AChE but also to evaluate whether structural modifications of the BPC-157 scaffold could influence enzyme binding and inhibitory activity.

The present study aimed to investigate, for the first time, the inhibitory effects of BPC-157 on acetylcholinesterase activity and to compare its activity with that of newly designed hybrid analogs, CIARA-1 and CIARA-2, using kinetic analysis. Particular attention was given to determining the mechanism of inhibition and evaluating inhibitory potency based on key kinetic parameters, including *K_m_*, *V*_max_, *K_i_*, and IC_50_.

## 2. Results and Discussion

The effects of BPC-157, CIARA-1, and CIARA-2 on enzymatic activity were investigated to determine whether they function as AChE inhibitors.

### 2.1. Analysis

**Figure 2:**
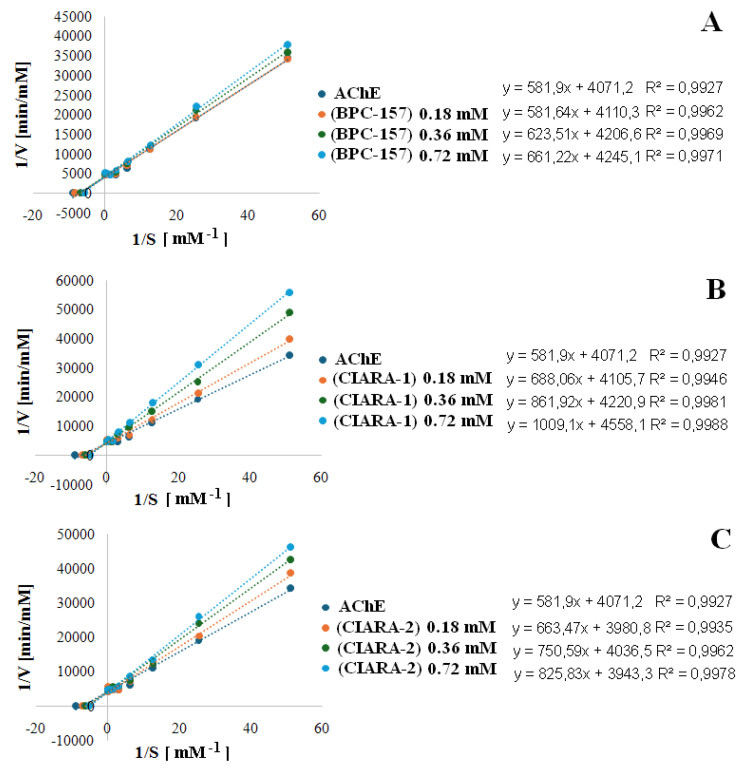
Lineweaver–Burk plots for AChE in the presence of BPC-157 (**A**), CIARA-1 (**B**), and CIARA-2 (**C**) at inhibitor concentrations of 0.18, 0.36, and 0.72 mM. Data points represent mean values ± SD from six independent experiments (n = 6). Enzymatic activity was measured spectrophotometrically at 412 nm using a modified Ellman assay with acetylthiocholine as the substrate in phosphate buffer (100 mM, pH 7.5) at 25 °C. Linear regression analysis was applied to visualize the inhibition mechanism.

In this work, experimental results were conducted to answer one key question: how do the tested compounds affect acetylcholinesterase activity? The lines in the plots represented the relationship between the reciprocal of the substrate concentration and the reciprocal of the reaction rate (Figure 2, Table 1).

The initial results showed that, in the absence of an inhibitor, the enzyme exhibited almost perfect linearity (AChE: r = 0.9927, slope = 581), yielding a Lineweaver–Burk slope of 89.90. However, as additional compounds were introduced into the system, the slopes of the lines began to change. Each of the tested inhibitors affected the reaction in a slightly different way. However, all shared a common characteristic: they increased the slope of the graph (BPC-157 = 89.92, CIARA-2 = 89.93, and CIARA-1 = 89.94 for a concentration of 0.72 mM), suggesting a decrease in the enzyme’s affinity for the substrate (Figure 2).

All graph lines intersected at the same point on the Y-axis (Figure 2), clearly indicating that the tested compounds act as competitive inhibitors. This pattern demonstrates competition with the substrate for the enzyme’s active site, without altering its maximum efficiency.

The deeper the data were analyzed, the more distinct the differences between the compounds became. CIARA-1 stood out from the others. Its presence caused the largest increase in *K_m_* (0.21 mM), indicating strong competition for the enzyme’s active site. CIARA-2 behaved similarly, though was slightly weaker (*K_m_* = 0.19 mM), while BPC-157 proved the least effective inhibitor of the three (*K_m_* = 0.17 mM at 0.72 mM) (Table 1, Figure 2).

Subsequent calculations confirmed the previous assumptions. The inhibition constants (*K_i_*) clearly indicated that CIARA-1 bound the enzyme most strongly. Its *K_i_* values were significantly lower (0.24) than those of the other compounds (for CIARA-2 = 0.29 and for BPC-157 = 0.48) (Table 1, Figure 2).

**Figure 3:**
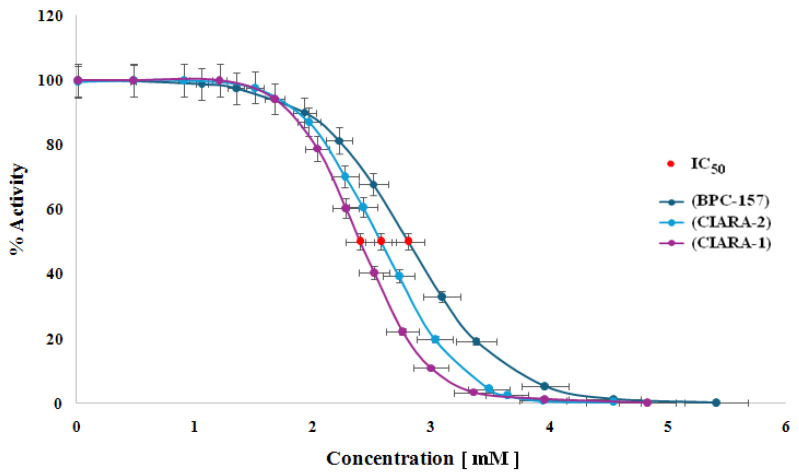
Graphical IC_50_ determination for inhibitors BPC-157, CIARA-2, and CIARA-1 toward acetylcholinesterase (AChE). Enzyme activity was determined using a modified Ellman spectrophotometric assay at 412 nm. IC_50_ values were calculated using nonlinear regression analysis based on a variable-slope sigmoidal dose–response model. Data are expressed as mean ± SD obtained from six independent experiments (n = 6).

A similar trend was observed in the graphical IC_50_ analysis presented in Figure 3. The inhibition curves clearly demonstrate that CIARA-1 exhibited the strongest inhibitory effect toward AChE, reaching 50% inhibition at the lowest concentration (IC_50_ = 2.52 mM). In contrast, CIARA-2 and BPC-157 required higher concentrations to achieve the same effect, with IC_50_ values of 2.73 mM and 2.80 mM, respectively (Table 1). The steeper inhibition profile observed for CIARA-1 further supports its higher affinity toward the enzyme and confirms its superior inhibitory potency compared to the remaining tested peptides.

Although CIARA-1 exhibited the lowest *K_i_* and IC50 values among the investigated peptides, the differences between the tested compounds should be interpreted with caution. The observed variations in kinetic parameters were relatively moderate and occurred within a narrow concentration range. Therefore, while the results suggest a trend toward stronger inhibitory activity for CIARA-1, they do not indicate a dramatic difference in biological potency between the investigated peptides.

Importantly, the primary objective of this study was not to identify highly potent acetylcholinesterase inhibitors comparable to clinically used drugs, but rather to demonstrate, for the first time, that BPC-157-derived hybrid peptides can modulate AChE activity and to characterize their mechanism of inhibition. In this context, the consistent observation of competitive inhibition for all tested compounds represents the most significant finding of the study.

Furthermore, the biological relevance of peptide-based inhibitors cannot be assessed solely based on *K_i_* or IC50 values. Additional factors, including peptide stability, pharmacokinetic behavior, tissue distribution, blood–brain barrier permeability, and potential multifunctional cytoprotective or anti-inflammatory effects, may substantially influence their therapeutic potential. These aspects were beyond the scope of the present in vitro study and should be addressed in future investigations.

Consequently, the present results should be interpreted primarily as a proof of concept demonstrating that rational structural modification of BPC-157 can influence interactions with acetylcholinesterase and modulate inhibitory activity, rather than as evidence of major potency differences between the tested analogs.

Statistical analysis confirmed significant differences in inhibitory potency between the investigated peptides. In particular, CIARA-1 exhibited significantly lower *K_i_* and IC50 values than BPC-157 (*p* < 0.05), indicating stronger inhibitory activity against AChE. CIARA-2 also demonstrated significantly stronger inhibition than BPC-157, although its activity remained lower than that of CIARA-1. These findings support the conclusion that structural modification of the parent peptide significantly influences enzyme binding affinity and inhibitory efficiency.

**Figure 4:**
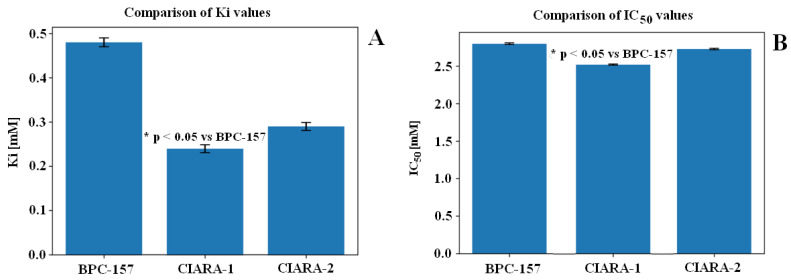
Statistical comparison of *K_i_* (**A**) and IC_50_ (**B**) values for BPC-157, CIARA-1, and CIARA-2. Data are presented as mean ± SD (n = 6). Statistical analysis was performed using one-way ANOVA followed by Tukey’s multiple comparisons test. * *p* < 0.05 vs. BPC-157.

Ultimately, the obtained results clearly demonstrated that all three compounds demonstrate a common mechanism of action but differ in their AChE inhibition potency. CIARA-1 emerged as the most potent inhibitor, clearly outperforming CIARA-2 and BPC-157 (Figure 4).

Research on acetylcholinesterase activity has long been a crucial element in the search for new therapeutic strategies, particularly in the context of neurodegenerative diseases. It was in this context that the idea arose to use three peptides to study AChE activity: the basic (starting) peptide BPC-157 and two derived hybrid structures, CIARA-1 and CIARA-2. These peptides were subjected to detailed kinetic analysis to uncover their potential as AChE enzyme inhibitors.

The initial results shed light on their mechanism of action. All three compounds behaved in an orderly manner, exhibiting characteristics of competitive inhibitors: an increase in the Michaelis–Menten constant (*K_m_*) with an unchanged maximum reaction velocity (*V*_max_). This pattern was consistently observed for all compounds tested. This indicates that the inhibitors directly compete with the substrate (acetylthiocholine) for binding to the active site of AChE, without affecting catalytic turnover [20,21]. The intersection of the Lineweaver–Burk plots on the y-axis further confirmed the classical mechanism of competitive inhibition.

Subsequent studies showed that *K_m_* values increased, while *V*_max_ values remained unchanged, consistent with competitive binding within the catalytic cleft of AChE [22]. However, despite their common mechanism of action, the three compounds were not equally potent. Of the three inhibitors tested, CIARA-1 clearly stood out as the most active. CIARA-2 ranked as intermediate, while BPC-157, although active, remained the weakest inhibitor in this comparison.

The differences in inhibition potency were further confirmed by the *K_i_* and IC_50_ values, with CIARA-1 demonstrating the lowest *K_i_* value, indicating the strongest binding affinity for AChE, followed by CIARA-2 and BPC-157. This trend is consistent with the current understanding that lower *K_i_* and IC_50_ values reflect stronger enzyme–inhibitor interactions and higher pharmacological potential [23]. It is important to note that the increase in the slope of the Lineweaver–Burk plots with increasing inhibitor concentration further emphasized the concentration-dependent nature of this interaction.

The most pronounced effect observed for CIARA-1 suggests an increased interaction with the AChE active site, potentially involving both the catalytic active site (CAS) and the peripheral anionic site (PAS). Modern structural and computational studies emphasize the importance of bipartite binding for increased inhibitory potential and multifunctional activity [24].

The obtained results also place the inhibitory potency of the studied peptides into a realistic pharmacological context. Compared to well-known drugs such as donepezil, physostigmine, and rivastigmine, the tested peptides are significantly weaker. Their affinity for the enzyme is many times lower, and their IC_50_ values are significantly higher.

The affinity of BPC-157, CIARA-1, and CIARA-2 for AChE was 6857, 3429, and 4143 times lower, respectively than that of donepezil (*K_i_* = 0.070 µM); 16,000, 8000, and 9667 times lower, respectively, than that of physostigmine (*K_i_* = 0.030 µM); and 32, 16, and 19 times lower, respectively, than that of rivastigmine (*K_i_* = 15 µM) [25]. Half-maximal inhibitory concentration (IC_50_) for BPC-157, CIARA-1, and CIARA-2 was 4.2 million, 3.7 million, and 4.0 million times higher, respectively, than that of donepezil (IC_50_ ≈ 0.0067 μM); 6.5 million, 5.8 million, and 6.3 million times higher, respectively, than that of rivastigmine (IC_50_ ≈ 0.0043 μM); and 4.2 million, 3.8 million, and 4.1 million times higher, respectively, than that of physostigmine (IC_50_ ≈ 0.00067 μM) [26].

The present study focuses primarily on the comparative analysis of the experimentally determined kinetic parameters obtained under identical assay conditions for BPC-157, CIARA-1, and CIARA-2. Among the investigated peptides, CIARA-1 consistently demonstrated the strongest inhibitory activity, reflected by the lowest *K_i_* and IC_50_ values, followed by CIARA-2 and BPC-157. These findings are fully supported by both kinetic analysis and molecular docking results, indicating enhanced interactions of CIARA-1 with the acetylcholinesterase binding region. It should be noted that the comparison with clinically used AChE inhibitors such as donepezil and rivastigmine is based on literature-reported values obtained under different experimental conditions, assay protocols, and enzyme sources. At this stage, the primary objective of our work was to determine whether BPC-157 and its hybrid analogs exhibit measurable AChE inhibitory activity and to characterize their inhibition mechanism using a consistent experimental framework. The inclusion of clinically established inhibitors as direct positive controls is an important aspect that we plan to incorporate in future studies aimed at a more comprehensive pharmacological evaluation.

Notably, the experimental findings are consistent with molecular docking predictions, which indicated stronger binding of CIARA-1 than of CIARA-2 or BPC-157. Such agreement between in silico and in vitro approaches is increasingly emphasized in recent drug discovery strategies, where computational modeling plays a crucial role in identifying and optimizing AChE inhibitors [27].

In a broader context, this research is part of the ongoing search for new AChE inhibitors for the treatment of Alzheimer’s disease.

Inhibition of AChE enhances cholinergic neurotransmission and partially compensates for the loss of cholinergic neurons, which is a hallmark of cognitive decline [28]. Recent studies also indicate that AChE is a multifunctional enzyme involved in β-amyloid aggregation via its peripheral anionic site, making it an attractive target for multi-target-directed ligands (MTDLs) [4].

In recent years, there has been growing interest in peptide-based AChE inhibitors due to their potential advantages, including greater selectivity, reduced toxicity, and the ability to target multiple binding sites simultaneously. Structure–activity relationship studies confirm that peptide sequences can be optimized to enhance binding affinity and inhibitory activity toward AChE [29,30]. In this context, the superior activity of CIARA-1 may be attributed to favorable structural features that enhance its interaction with the enzyme.

Despite these promising findings, the study has several limitations. The experiments were conducted under in vitro conditions using purified enzyme systems, which do not fully reflect physiological complexity. Factors such as metabolic stability, blood–brain barrier permeability, and peptide degradation may significantly affect in vivo efficacy. Recent literature emphasizes the importance of integrating biochemical, computational, and in vivo approaches to fully evaluate the therapeutic potential of novel AChE inhibitors [27,28].

However, several pharmacokinetic and physicochemical limitations characteristic of peptide-based compounds must be considered. The investigated peptides differ substantially from clinically used small-molecule AChE inhibitors (such as donepezil, physostigmine, and rivastigmine) in terms of molecular size, polarity, conformational flexibility, and susceptibility to enzymatic degradation, all of which may significantly influence their in vivo efficacy.

Preliminary assessment of physicochemical properties suggests that BPC-157, CIARA-1, and CIARA-2 exhibit relatively high polarity and limited lipophilicity due to the presence of multiple peptide bonds and ionizable amino acid residues. In particular, the positively charged arginine-containing C-terminal fragment introduced into CIARA-1 and CIARA-2 may enhance electrostatic interactions with AChE; however, it may simultaneously reduce passive diffusion across biological membranes, including the blood–brain barrier (BBB). Moreover, peptide-based structures are generally susceptible to proteolytic degradation in plasma and gastrointestinal environments, potentially limiting systemic bioavailability and metabolic stability.

Nevertheless, several structural features of the investigated compounds may partially improve peptide stability. For instance, the presence of proline-rich motifs may reduce conformational flexibility and increase resistance toward proteolytic cleavage.

Numerous modern strategies have been proposed to improve the pharmacokinetic profile and blood–brain barrier (BBB) penetration of therapeutic peptides, including cyclization, incorporation of D-amino acids, PEGylation, lipidation, nanoparticle-based delivery systems, intranasal administration, and conjugation with cell-penetrating peptides or BBB shuttle sequences [31,32,33,34,35,36]. In particular, multifunctional hybrid peptide systems combined with nanocarrier-based delivery approaches are increasingly recognized as promising strategies for central nervous system therapeutics [33,34,35,36].

Therefore, although the present study demonstrates in vitro AChE inhibitory potency, the investigated hybrid peptides may constitute valuable lead structures for further optimization to improve stability, biodistribution, and brain accessibility. Future studies should include detailed ADMET (Absorption, Distribution, Metabolism, Excretion, Toxicity) profiling, plasma stability assays, permeability studies, and in vivo pharmacokinetic evaluation to assess their therapeutic potential fully.

In conclusion, BPC-157, CIARA-1, and CIARA-2 act as reversible competitive inhibitors of AChE, with CIARA-1 demonstrating the highest potency and binding affinity. These findings contribute to the growing body of research on peptide-based cholinesterase inhibitors and support further investigation into hybrid peptide structures as potential therapeutic agents.

### 2.2. Molecular Docking

Molecular docking was performed to rationalize the experimentally observed inhibition mechanism and to characterize the binding modes of BPC-157, CIARA-1, and CIARA-2 toward acetylcholinesterase. The results clearly indicate that all investigated peptides bind preferentially to the enzyme conformation represented by the competitive model (PDB: 4EY6), consistent with the inhibition mechanism derived from kinetic analysis.

The applied docking strategy, based on comparing ligand binding to enzyme conformations associated with competitive and non-competitive inhibition models, follows our recently established and validated approach reported in earlier studies on small-molecule inhibitors [1,37]. In those works, the methodology successfully correlated molecular binding preferences with experimentally determined inhibition mechanisms. In the present study, this framework is extended to a new class of ligands, namely peptide-based compounds, enabling direct comparison of their binding behavior within the same validated model.

**Figure 5:**
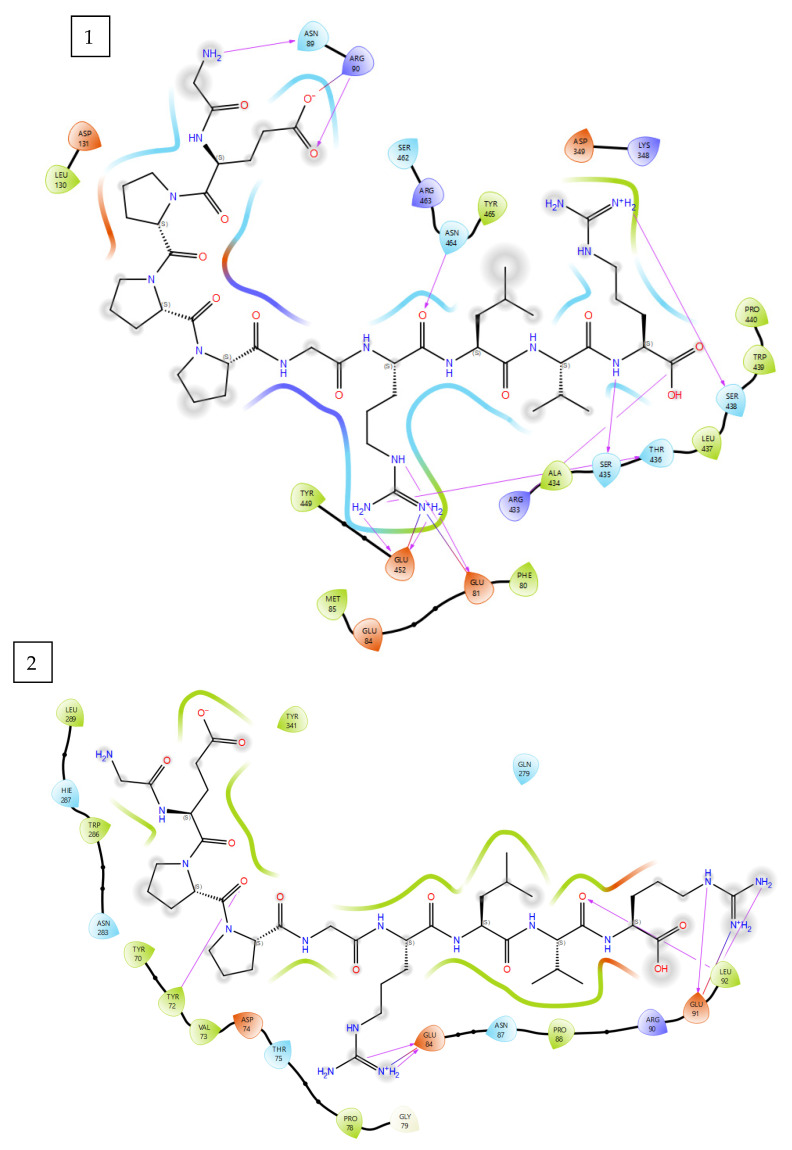

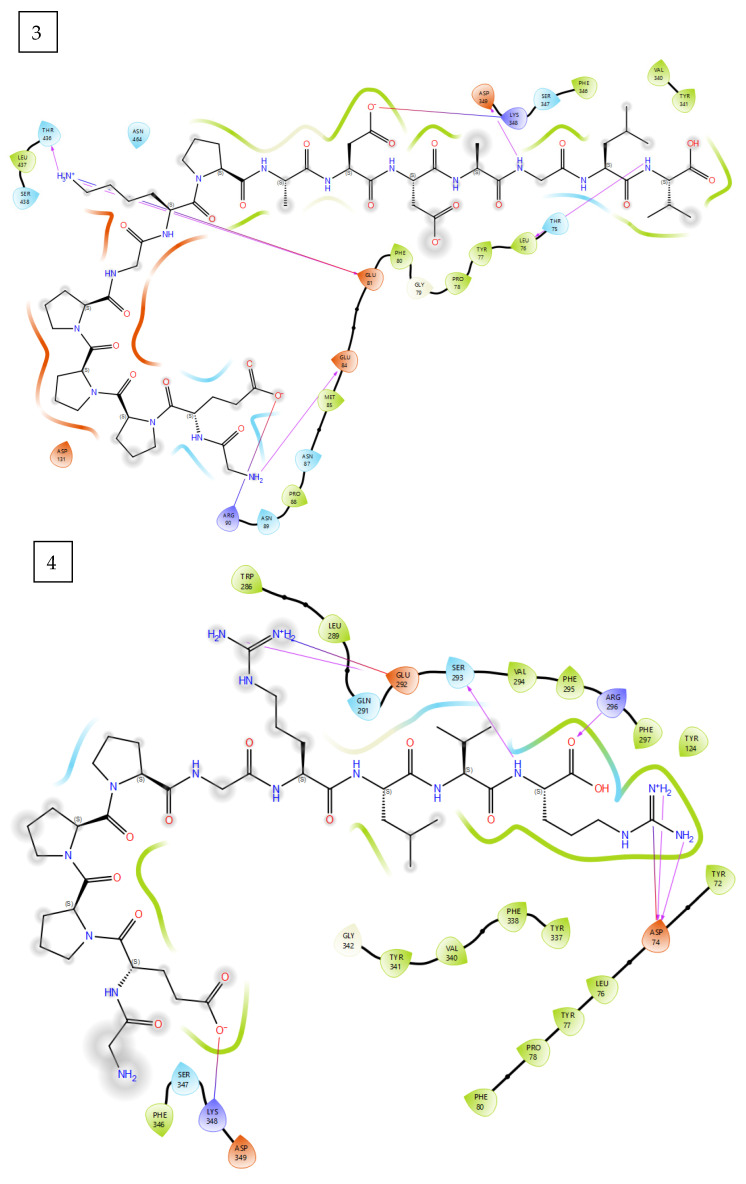

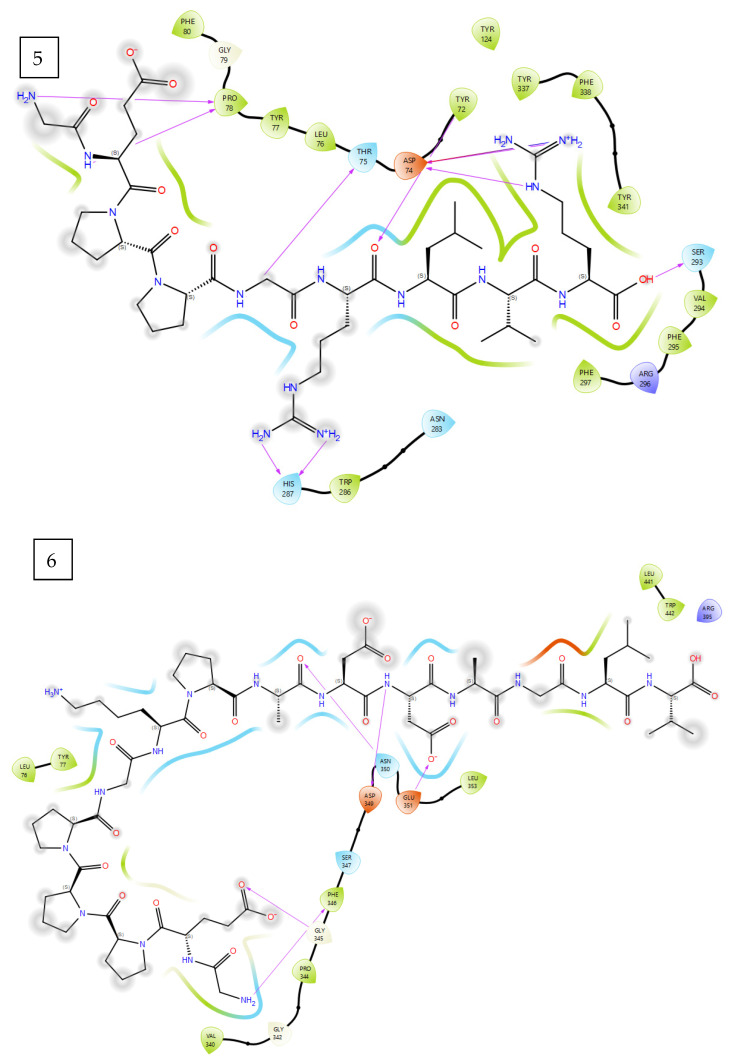
Binding modes of CIARA-1 (models 1 and 4), CIARA-2 (models 2 and 5), and BPC-157 (models 3 and 6) with AChE obtained from molecular docking. A description of the models (1–6) is presented in Table 2.

In the competitive model, all ligands adopt extended conformations and establish multiple stabilizing interactions with the protein environment, as illustrated in the interaction diagrams (Figure 5). The corresponding three-dimensional binding models for the competitive inhibition model are provided in Supplementary Figures S1–S3. In particular, numerous hydrogen bonds (marked by directional arrows in the figures) are formed between the peptide backbone and side chains and the surrounding amino acid residues. For CIARA-1, the interaction network is the most extensive, involving several simultaneous hydrogen bonds distributed along the ligand, which results in strong anchoring within the binding region. CIARA-2 exhibits a similar binding mode; however, the number and spatial distribution of hydrogen bonds are slightly reduced, leading to a less optimal stabilization pattern. In contrast, BPC-157 forms fewer interactions overall, and its binding appears more fragmented, which is consistent with its lower affinity.

**Table 2:** Molecular docking results for the complexes formed between CIARA-1, CIARA-2, BPC-157, and human AChE.

Protein PDB Code	Co-Crystallized Inhibitor	Docked Ligand	Model	GLIDE XP Score	MM-GBSA [kcal/mol]
4EY6	Competitive galantamine	CIARA-1	1	−9.715	−54.346
		CIARA-2	2	−9.365	−52.481
		BPC-157	3	−8.540	−38.971
7XN1	Non-competitive tacrine	CIARA-1	4	−7.277	−33.873
		CIARA-2	5	−7.261	−32.472
		BPC-157	6	−5.828	−24.281

The calculated binding energies fully support these qualitative observations. CIARA-1 shows the most favorable MM/GBSA value, followed by CIARA-2, while BPC-157 exhibits a significantly less favorable energy profile. This trend reflects the extent and organization of intermolecular interactions visible in the corresponding diagrams, where a more continuous, cooperative interaction network corresponds to stronger binding.

In the non-competitive model (PDB: 7XN1), all ligands exhibit markedly weaker, less organized binding patterns. The corresponding three-dimensional binding models for the non-competitive inhibition model are provided in Supplementary Figures S4–S6. As shown in the interaction diagrams, the number of hydrogen bonds is reduced, and the interactions are more localized rather than distributed along the entire ligand. The peptides adopt less favorable orientations and do not establish an extended interaction network comparable to that observed in the competitive model. Consequently, the calculated binding energies are significantly less favorable for all compounds in this conformation.

Overall, the docking results consistently demonstrate that the investigated peptides preferentially interact with the enzyme, as predicted by the competitive model, in full agreement with our previously reported structure–mechanism relationships [37,38]. The differences in binding affinity between CIARA-1, CIARA-2, and BPC-157 can be directly attributed to the number, distribution, and cooperativity of interactions observed in the docking poses, as clearly visualized in the interaction diagrams. These findings provide a structural basis for the experimentally observed differences in inhibitory potency.

## 3. Materials and Methods

### 3.1. Experimental

Peptides were synthesized manually by the solid-phase Fmoc/tBu methodology using a Kamush heating clamp (Kamush, Zblewo, Poland). Wang resin was used for the synthesis of BPC-157 (GEPPPGKPADDAGLV-OH), CIARA-1 (GEPPPGRLVR-OH), and CIARA-2 (GEPPGRLVR-OH) peptides. Fmoc deprotection was carried out at 60 °C (2 × 2 min) using a 20% (*v*/*v*) piperidine solution in DMF. Couplings were performed using an equimolar mixture of Fmoc-AA-OH, N,N-diisopropylcarbodiimide (DIC), and Oxyma Pure in a fivefold excess relative to the resin loading. The reactions were carried out at 60 °C for 15 min with constant shaking. Only the coupling of Fmoc-L-Arg(Pbf)-OH was performed without heating at room temperature (RT) for 60 min. The elongation of the peptide chain was carried out in consecutive cycles of deprotection and coupling. The peptides were cleaved from the resin using a mixture of trifluoroacetic acid (TFA), triisopropylsilane (TIS), phenol, and deionized water (92.5:2.5:2.5:2.5) for 1.5 h with agitation. The products were then precipitated with cold diethyl ether, dissolved in water, and lyophilized.

Purification was performed by RP-HPLC on a Phenomenex Gemini-NX C18 column (21.2 × 100 mm, 5.0 μm particle size, 110 Å pore size) using an Ecom preparative HPLC system (Ecom, Czech Republic). Water and acetonitrile, both containing 0.1% (*v*/*v*) TFA, were used as mobile phases. A linear gradient of 5–60% acetonitrile over 45 min was applied at a flow rate of 20.0 mL/min. Fractions were collected, analyzed by analytical HPLC, and combined to achieve a purity of >95%. The purity and identity of the peptides were confirmed by LC-MS (Figure S7) analysis using a Waters Alliance e2695 system equipped with Waters 2998 PDA and Acquity QDA detectors (software: Empower3, Waters, Milford, MA, USA). All analyses were carried out on a Halo C18 column (4.6 × 100 mm, 2.7 µm particle size, 90 Å pore size). Samples (5 µL) were analyzed using a linear gradient from 1% to 99% acetonitrile in deionized water over 10 min. The mobile-phase flow rate was 0.5 mL/min. Both eluents contained 0.1% (*v*/*v*) formic acid. Mass analysis and UV detection at 214 nm were employed.

The protocol outlined in our earlier study [38] was followed in conducting the studies. The reaction setup included pure AChE with or without the studied compounds (BPC-157, CIARA-1, and CIARA-2), 5,5′-dithio-bis-2-nitrobenzoic acid (DTNB) as the chromogenic reagent, and acetylthiocholine (ACth) as the substrate. A modified Ellman’s method was used to quantify the reaction product, 5-thio-2-nitrobenzoic acid (TNB). To determine baseline kinetics, enzyme activity was initially assessed across a range of substrate concentrations (0.39–200 mM ACth). The kinetic parameters and inhibition constants were then determined by conducting inhibition assays at three inhibitor concentrations (0.18, 0.36, and 0.72 mM) for each of the substances—BPC-157, CIARA-1, and CIARA-2—while varying the substrate concentration (Figure 2). The study began with a kinetic analysis, using three compounds—BPC-157, CIARA-1, and CIARA-2—and subjecting them to a series of tests. Using steady-state data, Lineweaver–Burk plots were generated.

Human acetylcholinesterase was used at a final concentration of 0.01 U/mL in the reaction mixture. Before initiation of the enzymatic reaction, all assay components, including phosphate buffer (100 mM, pH 7.5), DTNB, substrate, and inhibitor solutions, were preincubated for 10 min at 25 °C to allow equilibration of the system and stabilization of enzyme–inhibitor interactions. The reaction was initiated by adding AChE, and absorbance changes were monitored continuously at 412 nm for 5 min.

IC50 values were determined by nonlinear regression of concentration–response curves generated from residual enzymatic activity measured at increasing inhibitor concentrations. The percentage of inhibition was calculated relative to control samples containing the enzyme without the inhibitor according to the following equation:

$$\%\;inhibition=\left(1-\frac{V_{i}}{V_{0}}\right)\times 100$$

where *Vi* represents enzyme activity in the presence of the inhibitor, and V0 corresponds to the control enzyme activity. IC_50_ values were subsequently calculated using a variable-slope sigmoidal dose–response.

The relatively high inhibitor concentrations used in this study were selected based on preliminary screening experiments indicating weak-to-moderate inhibitory potency of the investigated peptides toward AChE. Unlike clinically used small-molecule inhibitors, peptide-based compounds typically exhibit lower binding affinity due to their larger size, greater conformational flexibility, and limited penetration into the enzyme’s catalytic gorge. Therefore, millimolar concentration ranges were necessary to obtain measurable concentration-dependent inhibition and enable the reliable determination of kinetic parameters and IC_50_ values.

#### Statistical Analysis

All experiments were performed in six independent repetitions (n = 6), and the obtained results are expressed as mean ± standard deviation (SD). Enzyme kinetic parameters were determined using Lineweaver–Burk analyses based on averaged reaction rate values obtained at different substrate and inhibitor concentrations.

The normality of the experimental data distribution was verified using the Shapiro–Wilk test before comparative analysis. Homogeneity of variance was assessed using Levene’s test. Statistical differences between control AChE activity and inhibitor-treated systems (BPC-157, CIARA-1, and CIARA-2 at different concentrations) were evaluated using one-way analysis of variance (ANOVA), followed by Tukey’s multiple comparisons post hoc test.

To compare kinetic parameters (*K_m_* and *V*_max_) obtained at increasing inhibitor concentrations, a repeated-measures ANOVA was additionally applied to evaluate concentration-dependent effects within each peptide group. Differences in *K_i_* and IC_50_ values between the investigated compounds were analyzed using ordinary one-way ANOVA with post hoc Tukey correction.

Linear regression was used to fit Lineweaver–Burk plots and determine slopes, intercepts, and correlation coefficients (R^2^). Nonlinear regression was used to determine IC_50_ values and inhibition constants (*K_i_*). Goodness of fit was evaluated using the coefficient of determination (R^2^) and residual analysis. Statistical significance was accepted at *p* < 0.05.

### 3.2. Reagents and Chemicals

Sigma-Aldrich (Poznań, Poland) supplied human acetylcholinesterase (AChE; EC 3.1.1.7), acetylthiocholine iodide (ACthI), 5,5′-dithio-bis-2-nitrobenzoic acid (DTNB), and 5-thio-2-nitrobenzoic acid (TNB). All tested peptides, i.e., BPC-157, CIARA-1, and CIARA-2, were synthesized using solid-phase peptide synthesis (SPPS), as described above.

### 3.3. Instrumentation

A Shimadzu UV-1280 tabletop UV-Vis spectrophotometer (198–1100 nm) was used for kinetic measurements.

### 3.4. Preparation of Standards

Deionized water was used to create stock solutions of BPC-157, CIARA-1, and CIARA-2 (2 M). A total of 2.8918 g of ACthI was dissolved in 50 mL of phosphate buffer (100 mM, pH 7.5) to create a 200 mM ACthI stock solution. Working solutions ranging from 0.39 to 200 mM were prepared using serial dilutions of ACthI.

### 3.5. Sample Preparation

A phosphate buffer (100 mM, pH 7.5) was used to dissolve AChE, which was then aliquoted and kept at −20 °C. Aliquots were frozen, and enzyme activity was confirmed before each experiment. The Ellman method [39], modified by Kaizer et al. [40], was used to quantify enzyme activity. Before adding AChE to initiate the reaction, reaction mixtures containing phosphate buffer, ACthI, inhibitor solution (BPC-157, CIARA-1, and CIARA-2), DTNB, and water were incubated at 25 °C. Spectrophotometric monitoring of product production was conducted at 412 nm.

### 3.6. Molecular Modeling

#### 3.6.1. Ligand Preparation

The structures of BPC-157, CIARA-1, and CIARA-2 were prepared using the LigPrep tool implemented in the Schrödinger Suite (Maestro, version 2023-3). The OPLS4 force field was applied during ligand preparation. Possible protonation states at physiological pH (7.5 ± 1.0) were generated using the Epik module to account for relevant ionization states of peptide functional groups. Geometry optimization was performed before docking.

#### 3.6.2. Protein Preparation

The crystal structures of human acetylcholinesterase (AChE) co-crystallized with a competitive inhibitor (PDB ID: 4EY6) and a non-competitive inhibitor (PDB ID: 7XN1) were obtained from the Protein Data Bank. Structures were processed using the Protein Preparation Wizard. The preparation procedure included adding hydrogen atoms, assigning bond orders, and optimizing hydrogen-bonding networks. Protonation states of ionizable residues were determined using the PROPKA algorithm at pH 7.5. The structures were subsequently minimized using the OPLS4 force field until the RMSD of heavy atoms reached 0.3 Å.

#### 3.6.3. Docking Protocol

Docking grids were generated for each protein structure based on the position of the co-crystallized ligand, defining the binding region corresponding to competitive and non-competitive inhibition models. The inner and outer grid boxes were set to 15 × 15 × 15 Å and 25 × 25 × 25 Å, respectively. Molecular docking was performed using the Glide module (Schrödinger Suite) in SP-peptide mode, which is specifically optimized for flexible peptide ligands. This protocol enables enhanced sampling of peptide conformations and improved treatment of backbone flexibility compared to standard docking procedures. For each ligand, multiple docking poses were generated, and the best-ranked conformations were selected based on Glide score and visual inspection of interaction patterns. Epik state penalties were excluded from the final scoring.

#### 3.6.4. MM/GBSA Calculations

Binding free energies (ΔG_bind_) were calculated using the MM/GBSA method implemented in the Prime module (Schrödinger Suite). The calculations were performed for the selected docking poses using the OPLS-2005 force field and the VSGB implicit solvent model, in accordance with the standard Schrödinger workflow.

Binding free energies were obtained according to:

$$\Delta G_{\mathrm{bind}}=G_{\mathrm{complex}}^{\mathrm{opt}}-(G_{\mathrm{protein}}^{\mathrm{opt}}+G_{\mathrm{ligand}}^{\mathrm{opt}})$$

For each individual system (complex, protein, and ligand), the total free energy was calculated as the sum of molecular mechanics contributions, solvation terms, and entropic factors:

$$G=G_{\mathrm{int}}+G_{\mathrm{Coulomb}}+G_{\mathrm{vdW}}+G_{\mathrm{GB}}+G_{\mathrm{lipo}}-TS$$

In this expression, T represents the absolute temperature, and S corresponds to the configurational entropy. The molecular mechanics energy includes contributions from bonded interactions (bond stretching, angle bending, and torsional terms) and non-bonded electrostatic and van der Waals interactions. The solvation energy is divided into a polar component (G_GB_), calculated using a generalized Born approach, and a non-polar term (G_lipo_) estimated from the solvent-accessible surface area (SASA).

## 4. Conclusions

This study provides the first evidence that BPC-157 and its hybrid analogs, CIARA-1 and CIARA-2, modulate acetylcholinesterase (AChE) activity through a reversible, competitive inhibition mechanism. Kinetic analysis demonstrated concentration-dependent increases in *K_m_* without significant changes in *V*_max_, while molecular docking supported preferential binding within the enzyme’s catalytic region.

Among the investigated compounds, CIARA-1 exhibited the highest inhibitory activity, followed by CIARA-2 and BPC-157. However, the observed inhibitory potency remained relatively weak, with IC50 values in the millimolar range and substantially lower than those of clinically used AChE inhibitors such as donepezil or rivastigmine.

Therefore, the present findings should be considered preliminary and exploratory rather than directly translational. The results mainly demonstrate that rational structural modification of BPC-157-derived peptides can influence AChE binding and inhibition profiles, providing an initial basis for further structure–activity relationship studies.

Importantly, the consistency between kinetic and molecular modeling data supports the reliability of the observed trends and indicates that hybrid peptide design may serve as a useful framework for future optimization. Further studies are required to improve inhibitory potency and to evaluate physicochemical, pharmacokinetic, and biological properties under more physiologically relevant conditions before any therapeutic relevance can be considered.

## Figures and Tables

**Table 1:** The analytical data describing the effects of acetylcholinesterase inhibition by BPC-157 and its hybrid analogs, CIARA-1 and CIARA-2.

		Slope of the Lineweaver–Burk Regression Line (Mean ± SD)	*K_m_* [mM] (Mean ± SD)	*V*_max_ [mM^−1^] × 10^−4^ (Mean ± SD)	*K_i_* (Mean ± SD)	IC_50_ [mM] (Mean ± SD)
	**AChE**	89.90 ±1.12	0.130 ± 0.010	2.46 ± 0.07		
**BPC-157**	**0.18 mM** **0.36 mM** **0.72 mM**	89.90 ± 1.15 89.91 ± 1.12 89.92 ± 1.12	0.120 ± 0.018 0.150 ± 0.016 0.170 ± 0.015	2.43 ± 0.06 2.41 ± 0.06 2.43 ± 0.05	0.480 ± 0.010	2.800 ± 0.010
**CIARA-1**	**0.18 mM** **0.36 mM** **0.72 mM**	89.92 ± 1.11 89.93 ± 1.13 89.94 ± 1.12	0.150 ± 0.017 0.170 ± 0.014 0.210 ± 0.014	2.40 ± 0.06 2.42 ± 0.05 2.40 ± 0.04	0.240 ± 0.009	2.520 ± 0.008
**CIARA-2**	**0.18 mM** **0.36 mM** **0.72 mM**	89.91 ± 1.15 89.92 ± 1.13 89.93 ± 1.14	0.140 ± 0.018 0.160 ± 0.015 0.190 ± 0.014	2.42 ± 0.07 2.41 ± 0.06 2.42 ± 0.06	0.290 ± 0.009	2.730 ± 0.009

Kinetic parameters describing acetylcholinesterase inhibition by BPC-157, CIARA-1, and CIARA-2. Data are expressed as mean ± SD obtained from six independent experiments (n = 6). Statistical analysis was performed using one-way ANOVA with Tukey’s multiple comparisons test. Differences were considered statistically significant at *p* < 0.05. The reported slope values were obtained from linear regression analysis of Lineweaver–Burk plots (1/V” versus “ 1/[S]) and were used for mechanistic interpretation of enzyme inhibition.

## Data Availability

The original contributions presented in this study are included in the article. Further inquiries can be directed to the corresponding author.

## Supplementary Materials

The following supporting information can be downloaded at: https://www.mdpi.com/article/10.3390/ijms27114984/s1.
