# Supplementary material for: BPC-157 and Its Novel Hybrid Analogs as Inhibitors of Acetylcholinesterase
Source: Int J Mol Sci. 2026 May 30;27(11):4984. doi: 10.3390/ijms27114984 (PMC13257072; doi:10.3390/ijms27114984)
Supplement: Supplementary file 1 [file ijms-27-04984-s001.zip › ijms-4334763-supplementary.pdf]

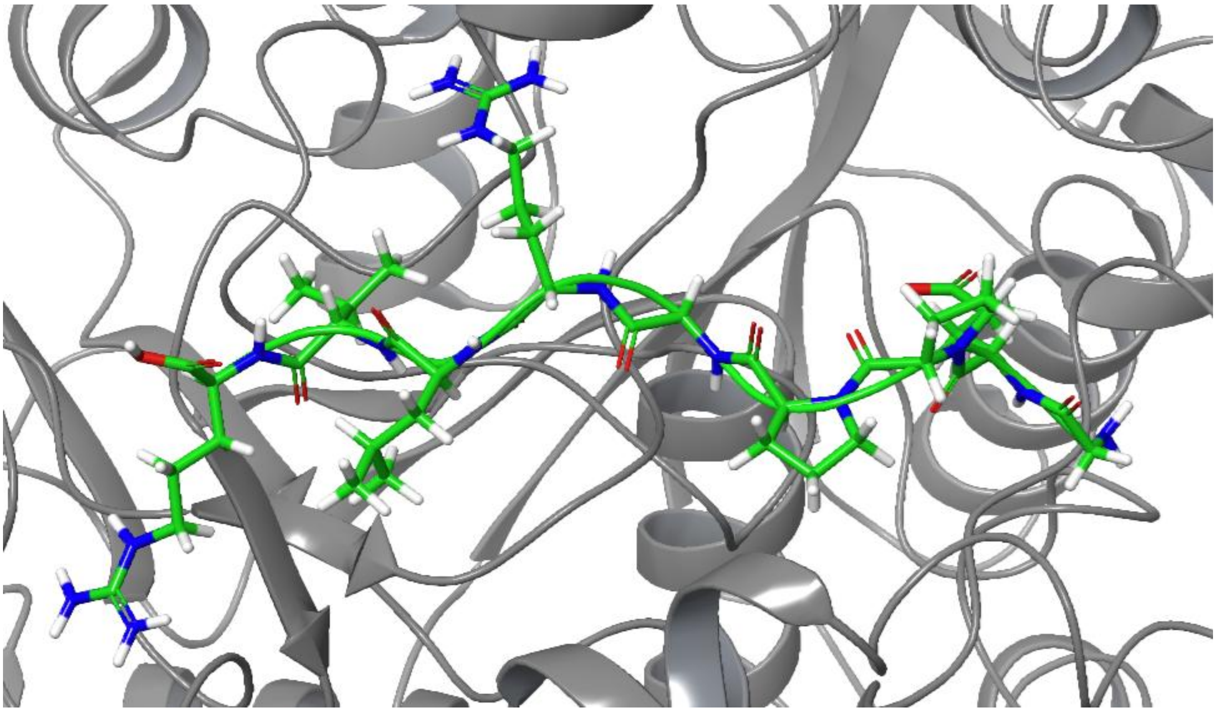

Figure S1. Three-dimensional binding model of CIARA-2 within the active site region of acetylcholinesterase in the competitive inhibition model (PDB ID: 4EY6). The peptide adopts an extended conformation and forms multiple stabilizing interactions with surrounding amino acid residues.

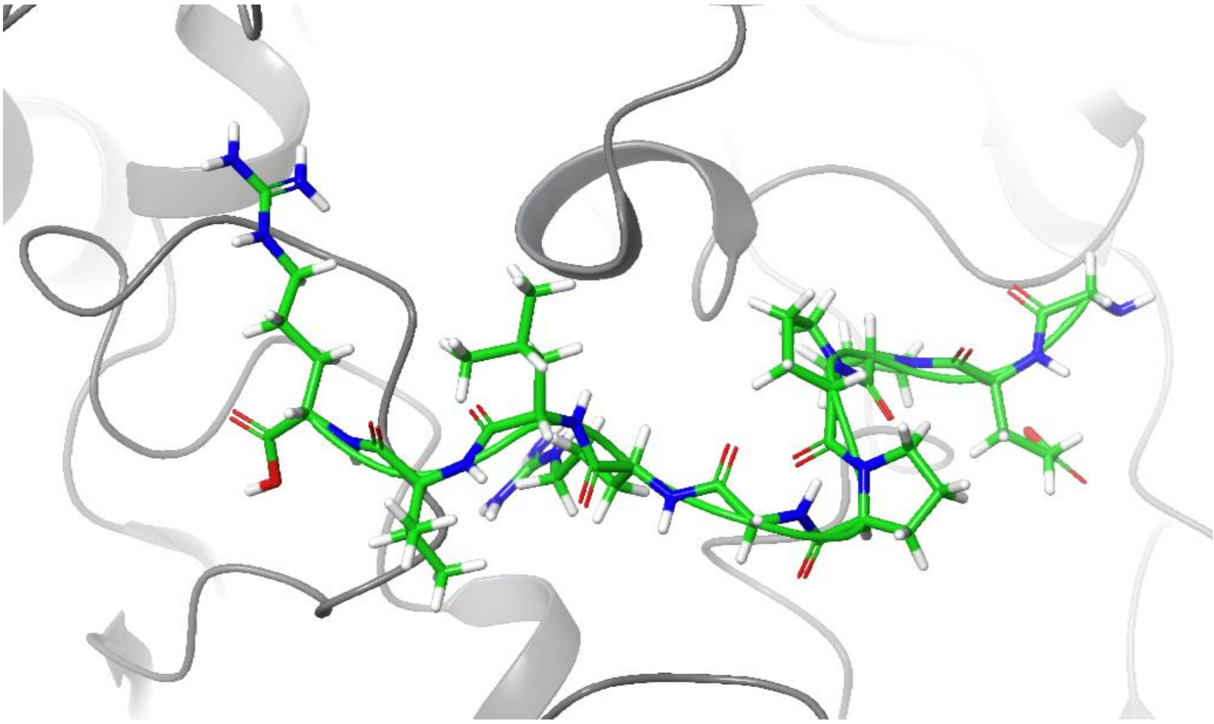

Figure S2. Three-dimensional binding model of CIARA-1 within the active site region of acetylcholinesterase in the competitive inhibition model (PDB ID: 4EY6). The peptide adopts an extended conformation and forms multiple stabilizing interactions with surrounding amino acid residues.

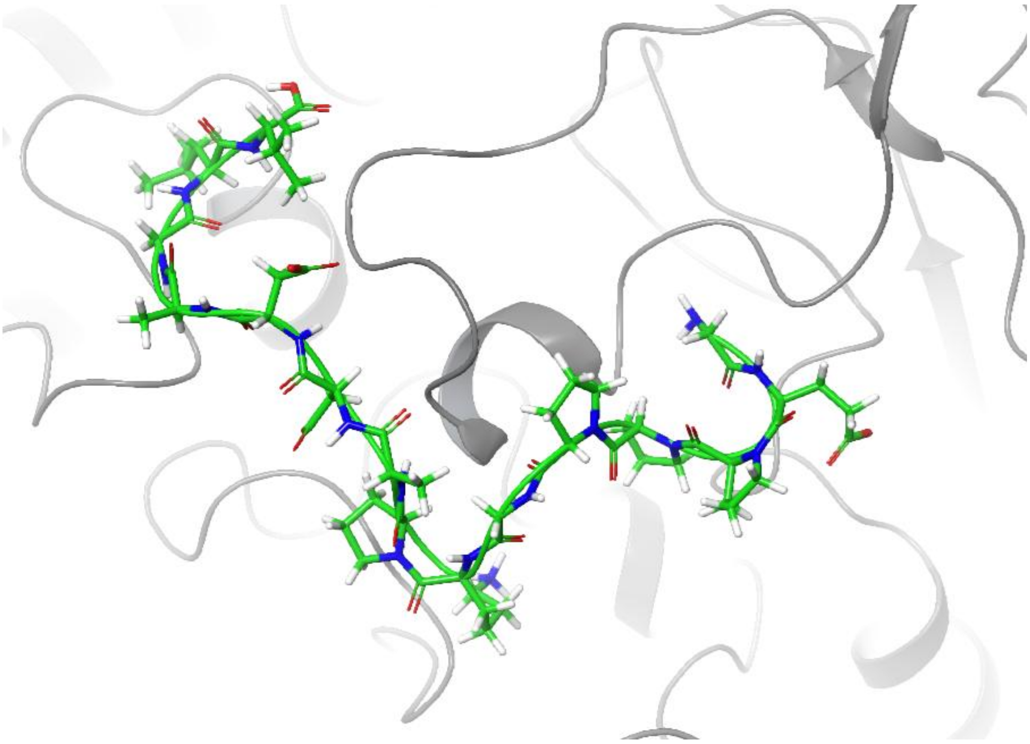

Figure S3. Three-dimensional binding model of BPC-157 within the active site region of acetylcholinesterase in the competitive inhibition model (PDB ID: 4EY6). The peptide forms fewer stabilizing interactions and displays a less cooperative binding network compared to the hybrid analogs.

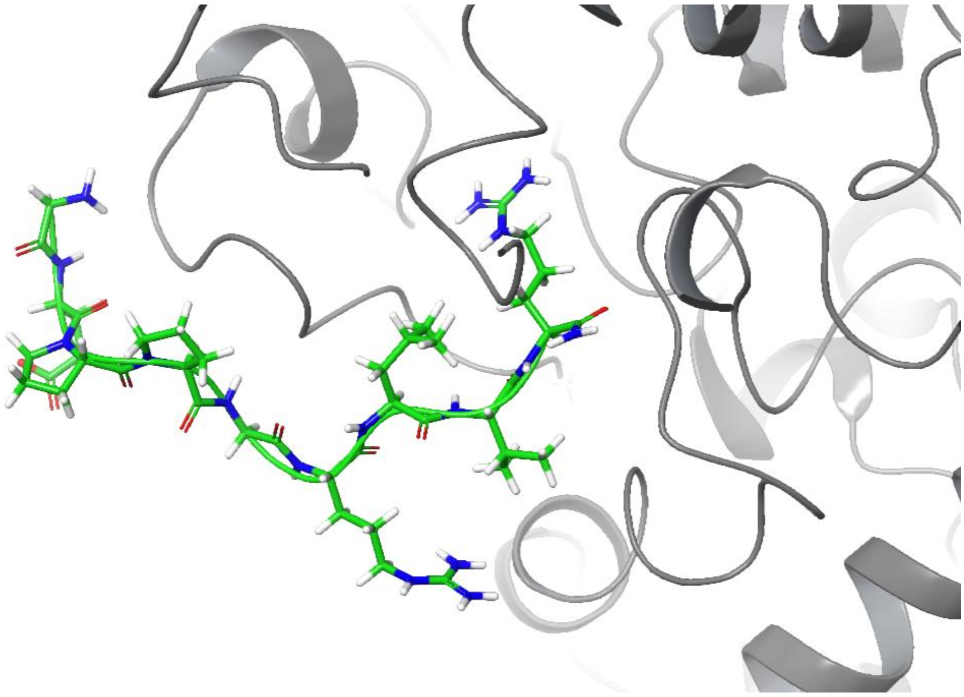

Figure S4. Three-dimensional binding model of CIARA-2 within acetylcholinesterase in the non-competitive inhibition model (PDB ID: 7XN1). A less organized interaction pattern and weaker stabilization are observed relative to the competitive binding model.

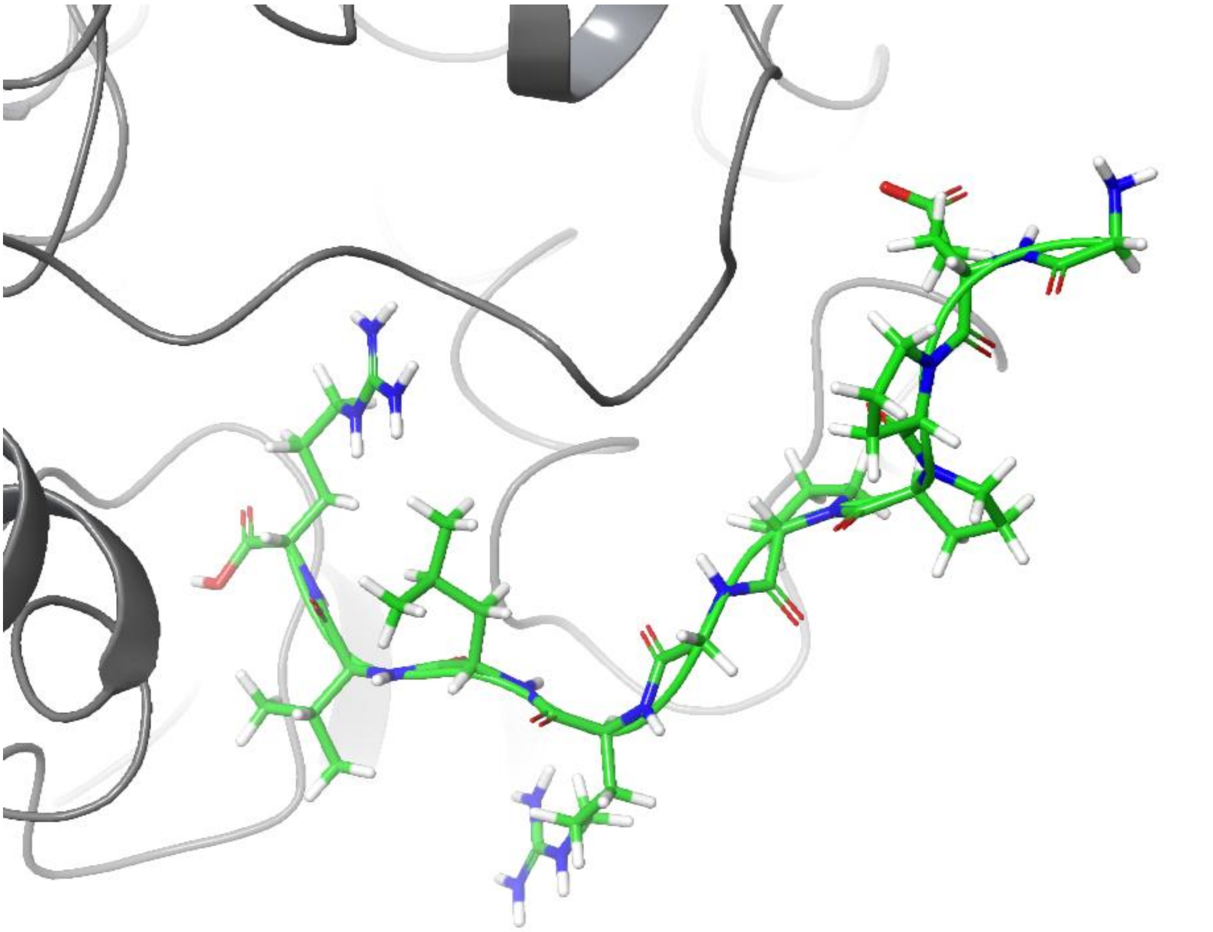

Figure S5. Three-dimensional binding model of CIARA-1 within acetylcholinesterase in the non-competitive inhibition model (PDB ID: 7XN1). A less organized interaction pattern and weaker stabilization are observed relative to the competitive binding model.

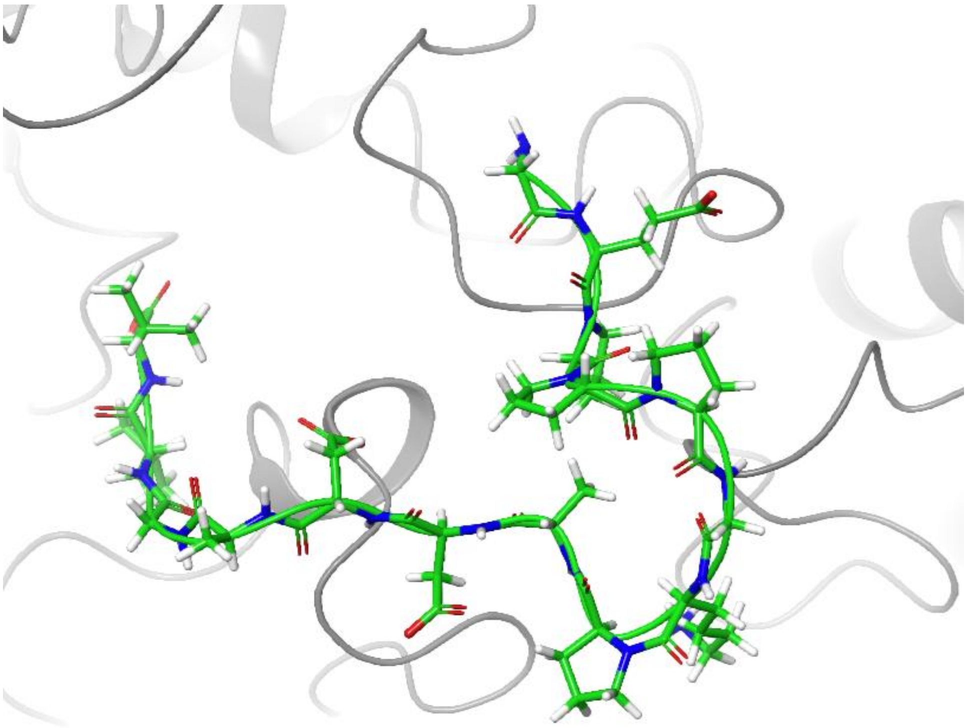

Figure S6. Three-dimensional binding model of BPC-157 within acetylcholinesterase in the non-competitive inhibition model (PDB ID: 7XN1). The ligand adopts a less favorable binding orientation characterized by limited intermolecular interactions.
